# Exposure of *Helicoverpa armigera* Larvae to Plant Volatile Organic Compounds Induces Cytochrome P450 Monooxygenases and Enhances Larval Tolerance to the Insecticide Methomyl

**DOI:** 10.3390/insects12030238

**Published:** 2021-03-12

**Authors:** Choufei Wu, Chaohui Ding, Shi Chen, Xiaoying Wu, Liqin Zhang, Yuanyuan Song, Wu Li, Rensen Zeng

**Affiliations:** 1 Key Laboratory of Vector Biology and Pathogen Control of Zhejiang Province, School of Life Sciences, Huzhou University, Huzhou 313000, China; wcf@zjhu.edu.cn (C.W.); fryo@163.com (X.W.); zlqwz@zjhu.edu.cn (L.Z.); 2 Guangdong Provincial Key Laboratory of Crop Genetic Improvement, Crops Research Institute, Guangdong Academy of Agricultural Sciences, Guangzhou 510640, China; dingchaohui123@gmail.com; 3 State Key Laboratory of Ecological Pest Control for Fujian and Taiwan Crops, College of Agriculture, Fujian Agriculture and Forestry University, Jinshan, Fuzhou 350002, China; yyuansong@fafu.edu.cn; 4 College of Materials and Energy, South China Agricultural University, Wushan, Guangzhou 510642, China; kkt@scau.edu.cn

**Keywords:** *Helicoverpa armigera*, plant volatile, herbivore-induced plant volatiles cytochrome P450 monooxygenases, insecticide tolerance, detoxification enzyme

## Abstract

**Simple Summary:**

Upon insect herbivory plants release herbivore-induced plant volatiles (HIPVs) to elicit either direct antiherbivore defenses by intoxicating the herbivore or indirect defenses by attracting natural enemies. Insect herbivores use sophisticated detoxification systems, including cytochrome P450 monooxygenases (P450s), to cope with plant defensive chemicals and insecticides. Many behaviors of insects are manipulated to a large extent by olfaction. However, few studies concern the role of olfaction in insect adaptation to xenobiotics. In this study we show that olfactory exposure of *Helicoverpa armigera*, a devastating pest of many important crop plants that has developed high levels of resistance to various insecticides, to four plant volatile compounds limonene, nerolidol, 2-heptanone and *cis*-3-hexenyl acetate dramatically increases larval tolerance to carbamate insecticide methomyl. Inhibition of P450s by piperonyl butoxide (PBO), a well-known P450 inhibitor, compromises the effects of volatile exposure. Exposure to the four volatiles upregulates multiple detoxification systems, including cytochrome P450s. Our findings indicate that induction of insecticide-detoxifying P450s by plant volatiles prior to actually inflicting plant damage may confer insect larvae more time to pre-activate insect detoxification systems prior to the ingestion or contact of insecticides, leading to increased larval tolerance to insecticides.

**Abstract:**

Plants release an array of volatile chemicals into the air to communicate with other organisms in the environment. Insect attack triggers emission of herbivore-induced plant volatiles (HIPVs). How insect herbivores use these odors to plan their detoxification systems is vital for insect adaptation to environmental xenobiotics. Here we show that the larvae of *Helicoverpa armigera* (Hübner), a broadly polyphagous lepidopteran herbivore, have the capacity to use plant volatiles as cues to upregulate multiple detoxification systems, including cytochrome P450 monooxygenases (P450s), for detoxification of insecticides. Olfactory exposure of the fifth instars to two terpene volatiles limonene and nerolidol, and two green-leaf volatiles 2-heptanone and *cis*-3-hexenyl acetate significantly reduced larval susceptibility to the insecticide methomyl. However, larval pretreatment with piperonyl butoxide (PBO), a known P450 inhibitor, neutralized the effects of volatile exposure. Furthermore, larval exposure to the four plant volatiles enhanced activities of P450 enzymes in midguts and fatbodies, and upregulated expression of CYP6B2, CYP6B6 and CYP6B7, P450s involved in detoxification of the insecticide. Larval exposure to 2-heptanone and limonene volatiles also enhanced activities of glutathione-s-transferase and carboxylesterase. Our findings suggest that olfactory exposure to HIPVs enhances larval insecticide tolerance via induction of detoxification P450s.

## 1. Introduction

Plants can synthesize a tremendous number of organic compounds. More than 200,000 natural organic compounds have been isolated and identified from plants. Some of these compounds are volatile organic compounds (VOCs), which are a class of low molecular weight lipophilic compounds. An amazing diversity of VOCs is released from plants to the surrounding environment at ambient temperatures. Over 1700 VOCs have been identified from 90 different plant families [1]. They mediate the interactions between plants and different species of organisms in the surrounding environment, from attracting pollinators [2] and seed transmission vectors to protecting themselves from herbivores, microbial pathogens and parasites [3,4,5,6]. By releasing VOCs, plants advertise to their surrounding organisms to establish a chemical communication network, which facilitate their interactions with the environment, and thereby affect the structure and function of ecosystems [7,8].

Plant emission of VOCs is often precisely timed and localized. In response to herbivore attack, plants increase emission of a complex blend of volatiles known as herbivore-induced plant volatiles (HIPVs) [9,10,11]. HIPVs mainly comprise green-leaf volatiles, terpenes including mono-, hemi- and sesquiterpenes, and some aromatic compounds. HIPVs are important media of plants for chemical communication and information exchange. These can serve either as direct antiherbivore defenses by intoxicating the herbivore or as indirect defenses by informing the location of the herbivore to predators and parasitoids [12,13]. HIPVs emitted from corn plants can directly repel and reduce food consumption by corn leaf aphids (*Rhopalosiphum maidis*) [14], and reduce *Spodoptera littoralis* infestations [15]. HIPVs exposure can increase vulnerability of the caterpillars of the noctuid *Spodoptera exigua* to natural entomopathogens [16]. HIPVs can also serve as communication signals between plants, recognized by the healthy parts of the same plant and neighboring plants, and induce systemic resistance in uninfected parts of the plant and in neighboring plants [17,18,19,20].

Plants synthesize and release HIPVs when they perceive a variety of herbivore behaviors such as crawling, feeding and oviposition. Numerous studies have showed beneficial effects of HIPVs towards host plants [13]. However, few studies address possible beneficial effects of HIPVs towards insect herbivores. Actually in plant–insect interactions insect herbivores do not act as passive victims. Many insect behaviors such as locating food, sexual partners and oviposition sites are manipulated to a large extent by olfaction [21]. Insect olfactory systems are able to rapidly and precisely respond to chemical stimuli including plant volatiles in their environment [22]. Insect olfactory environment is mostly made up of an amazing diversity of plant-emitted volatiles [23], which can be used by foraging insects to locate and recognize potential hosts. Olfactory manipulation is a promising approach for sustainable insect pest control [24]. A considerable amount of knowledge is available on how insects use volatile chemical signaling to locate and recognize their respective host species. However, in an enormous amount of research devoted to understanding how insects use olfaction to locate resources, the role of olfaction in insect detoxification has rarely been addressed.

Recently we demonstrated that larval olfactory perception of HIPVs elicits counter-defenses that enhance the ability of the tobacco cutworm *Spodoptera litura,* a polyphagous insect, to withstand host plant chemical defenses [25]. Here we examined the effect of exposure of two groups of plant volatiles (terpenoids and green leaf volatiles) on larval tolerance to the insecticides methomyl, and on activities of main detoxification enzymes and transcription of cytochrome P450 genes *CYP6B2*, *CYP6B6* and *CYP6B7* in *Helicoverpa armigera* (Lepidoptera: Noctuidae). *H. armigera* is one of the most destructive polyphagous insect pest with more than 200 host plant species. The insect has been demonstrated to develop high levels of resistance to an array of insecticides including carbamates, organophosphates and pyrethroids [26,27,28]. Cytochrome P450s play a key role in insecticide resistance in this species [28,29]. Terpenoids are the largest and most diverse class of secondary metabolites with many volatile constituents. Terpenoid VOCs are synthesized by the cytosolic mevalonic acid (MVA) and the plastidial methylerythritol phosphate (MEP) pathways. Green leaf volatiles (GLVs) are saturated and unsaturated C6/C9 aldehydes and alcohols that are usually synthesized in green organs of plants in response to wounding and insect herbivory, which confer fruits and vegetables with characteristic “fresh green” aroma [30,31]. The two groups of volatiles well represent HIPVs for investigating insect response to HIPVs in presence of xenobiotic insecticides. This study aimed to determine effects of olfactory exposure of *H. armigera* larvae to plant volatiles on insecticide resistance and a possible role of cytochrome P450s.

## 2. Materials and Methods

### 2.1. Chemicals

Methomyl (purity 98%) was obtained from Jiangsu Jinghong Chemical Co. Ltd. (Yancheng, China). Piperonyl butoxide (PBO, purity 98%) was purchased from Nanchang Yangpu Natural Essence and Spice Co. Ltd. (Nanchang, China). Limonene, nerolidol, 2-heptanone, cis-3-hexenyl acetate, NADPH, glycerol, 7-ethoxycoumarin (7-EC), 7-hydroxycoumarin (7-HC), phenylmethylsulfonyl (PMSF), dithiothreitol (DTT), phenylthiourea (PTU) and bovine serum albumin (BSA) were obtained from Sigma (St. Louis, MO, USA). Acetone was purchased from Shanghai Ling Feng Chemical Reagent Co. Ltd. (Shanghai, China).

### 2.2. Insects

The laboratory *H. armigera* larvae collected in Henan Province (China) were reared on an artificial diet as described by Gupta et al. [32] at 25 ± 1 °C, 50–70% RH, with a photoperiod of 14:10 (L: D). Five pairs of male and female adults were placed in a plastic mating cage with a 10% honey liquid. The eggs were collected and maintained in plastic cups for hatching. Newborn larvae (<24 h of age) were individually transferred to 20 mL plastic cups with lids containing artificial diet until usage or pupation.

### 2.3. Bioassay

Newly molted fifth instars of *H. armigera* were reared on artificial diets and concurrently exposed to different volatile organic compounds in sealed cups at a dose of 1 µL per cup for 48 h. A small ball of sterilized absorbent cotton was piled into a 3 µL pipette tube fixed on the lid of the cup. To avoid direct larval contact to the compounds the volatile (1 µL in liquid) was pipetted on the cotton in pipette tube and the cup lid was immediately sealed. The control group of larvae was fed on artificial diets without volatile exposure (CK). After 48 h exposure to volatiles the larvae were then treated with the insecticide methomyl (50 μg in 1 µL acetone per larva) on the pronotum. The larval mortality was recorded 24 h after methomyl treatment. Midguts and fatbodies of the larvae exposed to volatiles for 12, 24 and 48 h were collected and dissected for measurement of detoxification enzyme activities and transcription of detoxification genes. All bioassays were carried out with 3 replicates and 20 larvae for each replicate. To examine the potential involvement of P450 in volatile-induced insecticide tolerance, piperonyl butoxide (PBO, 3 µL), a known P450 inhibitor, was administered to larval pronotum 1 h before insecticide application in bioassays.

### 2.4. Activity Assays of Detoxification Enzymes GST, CarE and P450s

To determine possible effects of plant volatile exposure on detoxification enzymes, activities of glutathione-s-transferase (GST), carboxylesterase (CarE) and cytochrome P450 enzymes (P450s) were examined in the midguts and fatbodies of *H. armigera* larvae exposed to Limo, Nero, Hept and Hylac. Newly molted fifth instar larvae of *H. armigera* were exposed to the volatiles (1 µL per cup) for 12, 24 and 48 h, and then the midguts and fat bodies of 20 larvae per treatment were dissected on an ice plate and pooled. Each treatment had three replicates.

GST activity was evaluated spectrophotometrically using 1–chloro-2,4-dinitrobenzene (CDNB) and reduced glutathione (GSH) as standard substrates [33]. The tissues were homogenized in 2.4 mL 66 mM phosphate buffer (pH 7.0) containing 2 mM EDTA, 50 mM GSH 0.3 mL and 30 mM CDNB 0.1 mL. The homogenate was centrifuged at 10,000× *g* at 4 °C for 30 min. The enzyme solution was added to the cuvette and mixed. The reaction was monitored by measuring absorbance at 340 nm for 10 min. Enzyme activity of GST was calculated as described by Balakrishnan et al. [34]. The experiments were conducted with 3 biological samples and 3 technical replicates.

The carboxylesterase activity was determined by using α-naphthyl acetate (*α*-NA) as a substrate as described by Van Asperen [35] and modified by Chen et al. [36]. The midguts or fat bodies of 20 larvae were homogenized with 2 mL 0.04 M phosphate buffer on ice, then the homogenate was centrifuged at 12,000× *g* at −4 °C for 30 min, and the supernatant was diluted 50 times with 0.04 M phosphate buffer. The enzyme reaction mixture for CarE activity assay contained 50 μL of enzyme preparation, 450 μL of 0.04 M phosphate buffer at pH 7.0 and 3.6 mL of *α*-NA solution (0.3 mM). The absorbance of the hydrolysis product, α-naphthol, was measured at 600 nm.

For determination of P450 enzyme activity the midguts or fat bodies of 20 larvae were homogenized in 0.1 M phosphate buffer (pH 7.8) containing 1 mM EDTA, 0.1 mM DTT, 0.1 mM PMSF and 10% glycerol in an ice-cold mortar. The homogenate was centrifuged at 10,000× *g*, 4 °C for 20 min. The supernatant was collected and filtered through two layers of glass wool [37]. P450 enzyme activity was measured as described by Rose et al. [38] with some modification. Briefly, 90 µL of homogenate, 100 µL of 2 mM p-nitroanisole and 10 µL of 9.6 mM NADPH were poured into each well of the microplate. The absorbance was monitored at 405 nm at 30 °C for 10 min. Protein concentration was measured by the protocol of Bradford [39], with BSA as the standard.

### 2.5. qRT-PCR Analysis

To examine possible effects of plant volatile exposure on gene expression, qRT-PCR analysis was conducted to detect the transcript accumulation levels of three important cytochrome P450 monooxygenases (P450) genes *CYP6B2*, *CYP6B6* and *CYP6B7* potentially involved in detoxification of the insecticide in the fifth instars exposed to the four volatiles Limo, Nero, Hept and Hylac. After exposure to the volatiles for 12, 24 and 48 h, the midguts and fatbody were dissected for total RNA extraction and qRT-PCR. Total RNA was extracted from the midguts or fat bodies of 20 *H. armigera* larvae using Trizol Reagent kit (Invitrogen, Carlsbad, CA, USA). qRT-PCR was performed using SYBR Premix EX TaqTM (TaKaRa, Dalian, China) with a DNA Engine Opticon 2 Continuous Fluorescence Detection System (MJ Research Inc., Waltham, MA, USA). Each reaction (20 µL final volume) contained 1.0 µL cDNA, 10 µL SYBR Premix Ex TaqTM, 0.4 µL of each of forward and reverse primers specific for the mentioned genes, 0.4 µL Rox Reference Dye (50×) and 7.8 µL ddH_2_O. Reaction conditions for thermal cycling were 95 °C for 10 min, followed by 40 cycles of 10 s at 95 °C, 30 s at 60 °C and 15 s at 72 °C and the last step of 15 s at 95 °C, 30 s at 60 °C and 95 °C at 15 s. All assays were performed in triplicate and normalized using beta-actin as the internal control. Relative gene expression levels were calculated using the 2^−△△CT^ method [40] and the ABI 7300 analysis software. All primers used are listed in Supplementary Material Table S1.

### 2.6. Statistical Analysis

The statistical significance of differences (*p* ≤ 0.05) among treatments was determined by one-way analysis of variance (ANOVA) followed by a Tukey test for post-hoc comparison (*p* < 0.05) using SPSS software. The SPSS16.0 software program was used to perform all statistical analyses.

## 3. Results

### 3.1. Effect of Volatile Exposure on Larval Susceptibility to the Insecticide

**Figure 1:**
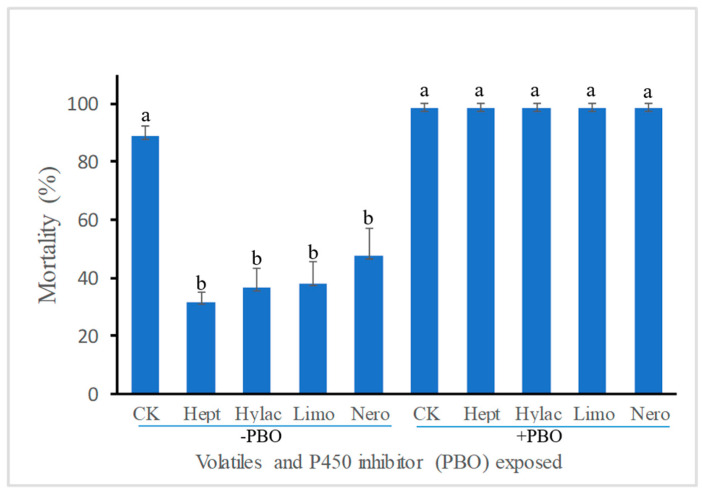
Mortality of *Helicoverpa armigera* caterpillars treated with methomyl after exposure to plant volatiles. Newly molted fifth instars were individually exposed to volatile 2-heptanone (Hept), *cis*-3-hexenyl acetate (Hylac), limonene (Limo) and nerolidol (Nero) at a dose of 1 µL per cup for 48 h in a sealed cup and then treated with methomyl (50 μg per caterpillar). No volatile exposure served as control (CK). Piperonyl butoxide (PBO, 3 µL), a known P450 inhibitor, was applied to larval pronotum 1 h before insecticide application. Mortality was recorded 24 h after methomyl treatment. Values are means + standard errors from three replicates with 20 caterpillars/replicate. Significant differences (*p* < 0.05 using ANOVA followed by a Tukey test for post-hoc comparison) among treatments in a group are indicated by different letters above the bars.

Olfactory exposure of the fifth instars to two terpene volatiles limonene (Limo) and nerolidol (Nero), and two green-leaf volatiles 2-heptanone (Hept) and *cis*-3-hexenyl acetate (Hylac) significantly reduced larval susceptibility to the insecticide methomyl (Figure 1). The larval mortality after methomyl treatment was reduced by 57.1%, 46.4%, 64.3% and 58.9% by exposure to Limo, Nero, Hept and Hylac, respectively. However, inclusion of piperonyl butoxide (PBO), a known P450 inhibitor, in the diet neutralized the effects of volatile exposure. The mortality of larvae pretreated with PBO was even higher than control regardless of volatile identity, suggesting a vital role of P450s in plant volatile-induced insecticide tolerance in *H. armigera*.

### 3.2. Effect of Volatile Exposure on Detoxification Enzymes

To determine possible effects of exposure to plant volatiles on metabolic detoxification of insecticide, activities of glutathione-s-transferase (GST), carboxylesterase (CarE) and cytochrome P450 enzymes (P450s) in midguts and fatbodies of *H. armigera* larvae exposed to volatile Limo, Nero, Hept and Hylac were evaluated. Surprisingly, exposure to all four selected plant volatiles obviously enhanced P450 activities in both midguts and fatbodies (Table 1). Exposure to Limo, Nero, Hept and Hylac increased P450 activities by 69.2%, 34.6%, 100% and 69.2% in the midguts and by 81.0%, 52.3%, 276.2% and 57.1% in the fatbodies, respectively. Furthermore, exposure to Hept and Limo also significantly increased activities of GST and CarE. However, exposure to Nero and Hylac did not significantly increase activities of GST and CarE. These results suggest that P450s play a more important role in plant volatile-induced insecticide tolerance in *H. armigera*.

### 3.3. Effect of Volatile Exposure on Transcript Levels of P450 Genes

**Figure 2:**
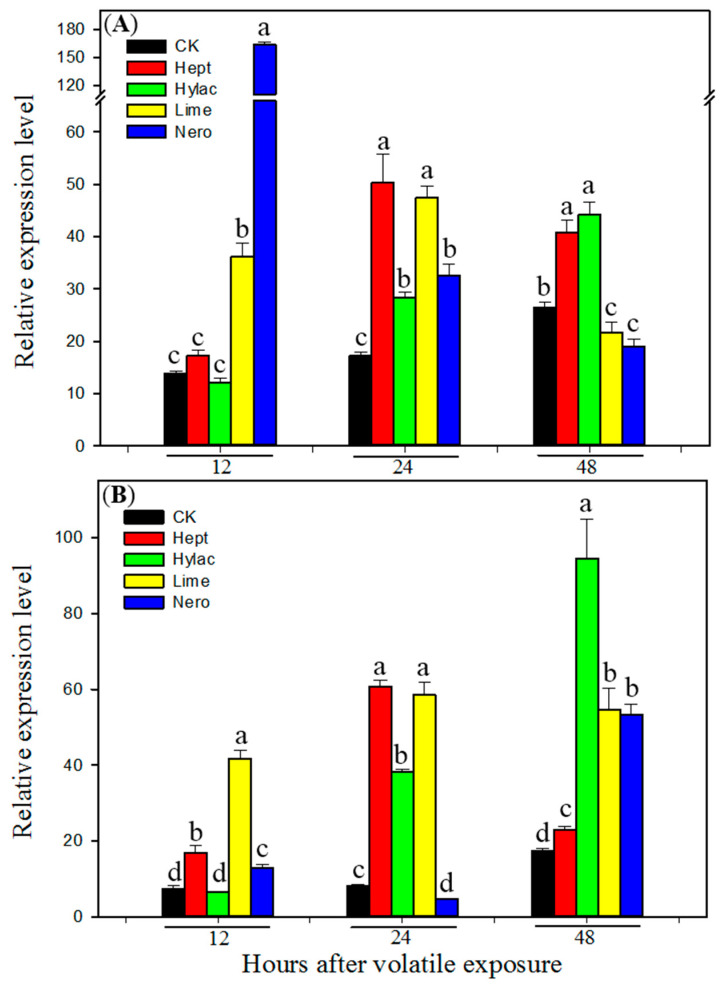
Transcript levels of *CYP6B2* in midguts (**A**) and fat bodies (**B**) of fifth instar larvae of *Helicoverpa armigera* exposed to plant volatiles. Newly molted fifth instars were individually exposed to volatile 2-heptanone (Hept), *cis*-3-hexenyl acetate (Hylac), limonene (Limo) and nerolidol (Nero) at a dose of 1 µL per cup for 12, 24 or 48 h, respectively in a sealed cup. Then the midguts and fat bodies were dissected. Total RNA was pooled from midguts or fat bodies of 20 fifth instar *H. armigera* larvae exposed to different plant volatile for 12, 24 and 48 h. Control larvae (CK) were fed with diet without plant volatile. Real-time qRT-PCR analysis was conducted to determine the relative transcript levels of the gene. Data shown are means ± SE derived from three biological repeats (*n* = 3). Different letters above bars indicate significant differences (*p* < 0.05 using ANOVA followed by a Tukey test for post-hoc comparison).

**Figure 3:**
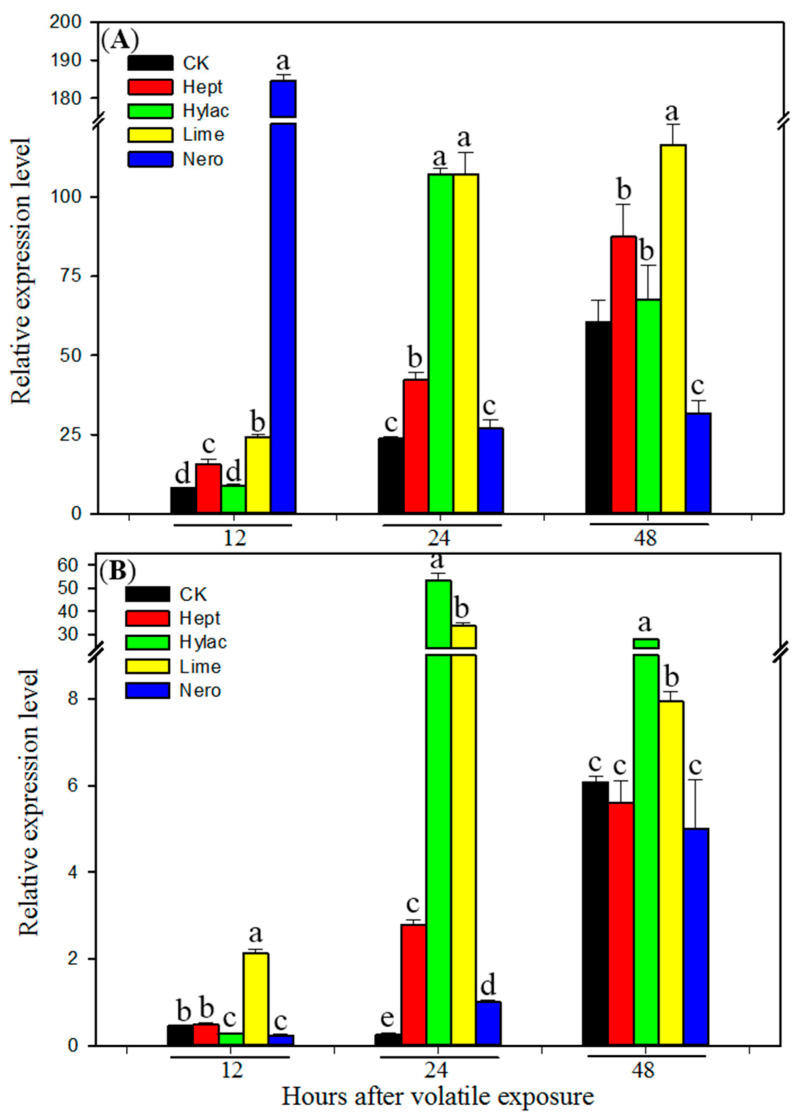
Transcript levels of *CYP6B6* in midguts (**A**) and fat bodies (**B**) of fifth instar larvae of *Helicoverpa armigera* exposed to plant volatiles. Newly molted fifth instars were individually exposed to volatile 2-heptanone (Hept), *cis*-3-hexenyl acetate (Hylac), limonene (Limo) and nerolidol (Nero) at a dose of 1 µL per cup for 12, 24 or 48 h, respectively in a sealed cup. Then the midguts and fat bodies were dissected. Total RNA was pooled from midguts or fat bodies of 20 fifth instar *H. armigera* larvae exposed to different plant volatile for 12, 24 and 48 h. Control larvae (CK) were fed with diet without plant volatile. Real-time qRT-PCR analysis was conducted to determine the relative transcript levels of the gene. Data shown are means ± SE derived from three biological repeats (*n* = 3). Different letters above bars indicate significant differences (*p* < 0.05 using ANOVA followed by a Tukey test for post-hoc comparison).

**Figure 4:**
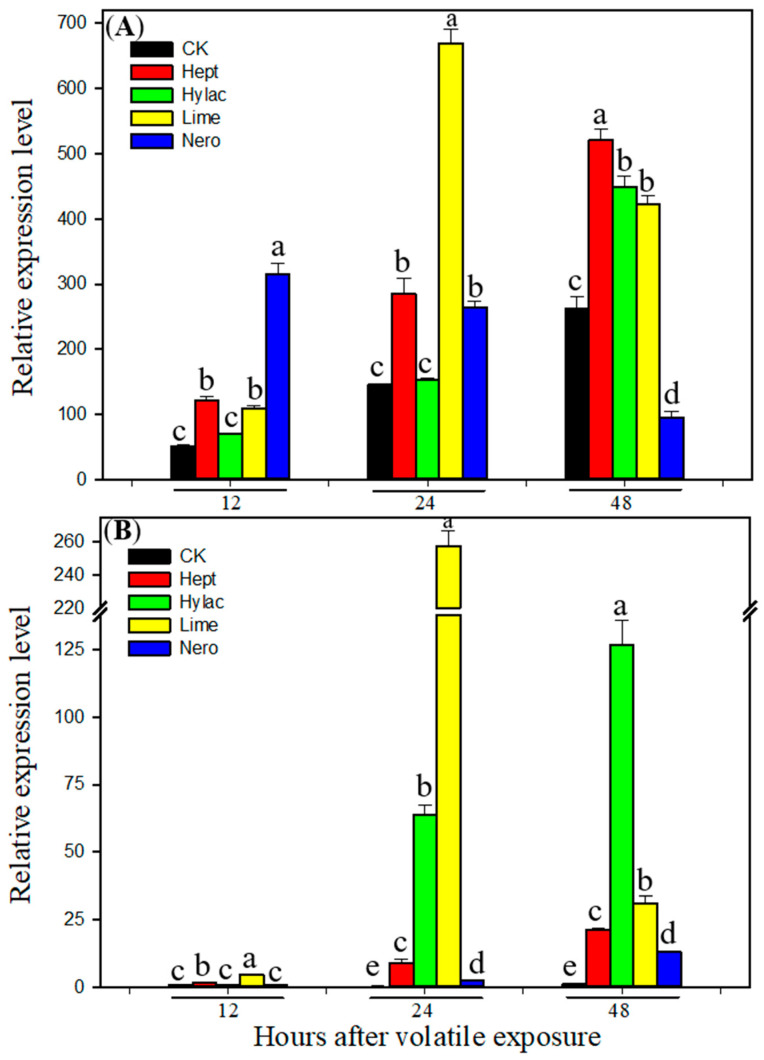
Transcript levels of *CYP6B7* in midguts (**A**) and fat bodies (**B**) of fifth instar larvae of *Helicoverpa armigera* exposed to plant volatiles. Newly molted fifth instars were individually exposed to vola-tile 2-heptanone (Hept), *cis*-3-hexenyl acetate (Hylac), limonene (Limo) and nerolidol (Nero) at a dose of 1 µL per cup for 12, 24 or 48 h, respectively in a sealed cup. Then the midguts and fat bodies were dissected. Total RNA was pooled from midguts or fat bodies of 20 fifth instar *H. ar-migera* larvae exposed to different plant volatile for 12, 24 and 48 h. Control larvae (CK) were fed with diet without plant volatile. Real-time qRT-PCR analysis was conducted to determine the rel-ative transcript levels of the gene. Data shown are means ± SE derived from three biological re-peats (*n* = 3). Different letters above bars indicate significant differences (*p* < 0.05 using ANOVA followed by a Tukey test for post-hoc comparison).

To determine whether plant volatiles enhance *H. armigera* tolerance to the insecticide by induction of P450 genes in, transcript levels of *CYP6B2*, *CYP6B6* and *CYP6B7* were examined by real-time qRT-PCR using RNA samples from midguts and fat bodies of newly molted fifth instars exposed to each of the four plant volatiles for 12, 24 and 48 h. All four volatiles induced expression of the three P450 genes in both midguts and fat bodies (Figure 2, Figure 3 and Figure 4). Nero showed the highest induction of all three P450 genes in the midguts 12 h after exposure, while the compound showed lower induction in the fat bodies and at other later time points. Limo showed the highest induction of all three P450 genes 24 h after exposure in both midguts and fat bodies. In general the larvae showed slower responses to green-leaf volatiles Hept and Hylac. Hept showed the highest induction of *CYP6B6* and *CYP6B7* 48 h after exposure in both midguts and fat bodies (Figure 3 and Figure 4), while Hylac showed the highest induction of *CYP6B2* and *CYP6B7* 48 h after exposure in the midguts and fat bodies (Figure 2 and Figure 4). All three P450 genes were induced by the four volatiles either in midguts or fat bodies 24 h after exposure. These results further confirm that P450s play an essential role of P450s in plant volatile-induced insecticide tolerance in *H. armigera*.

## 4. Discussion

The mediation of plant VOCs in signaling between plants and other organisms has been well documented. HIPVs have been shown to play many essential roles in plant–insect interaction [13,15,41]. They can either directly repel the insect herbivores, attract natural enemies predators and parasitoids to herbivore-attacked plants, or prime plant defense in uninfested parts of the plant and neighboring plants. On the other side herbivorous insects can develop multiple strategies to respond to the HIPVs. For instance, insect herbivores can use HIPVs as mate- and food-finding cues [42,43]. The lepidopteran moth of *Heliothis virescens* uses HIPVs emitted from tobacco plants to locate oviposition sites [44]. Methyl salicylate (MeSA), a volatile released from damaged soybean leaves, is a collective foraging signal for the larvae of *Loxostege sticticalis* [45]. *Spodoptera frugiperda*, an invasive insect pest in Africa and Asia, inhibits HIPVs emissions from maize [46]. *S. littoralis* caterpillars can utilize host-derived indole emissions to reduce their suitability and attractiveness to parasitoids by changing the smell of caterpillars [47]. HIPVs such as (E)-β-ocimene can also improve immune functions of *S. litura* [48].

Here we show that exposure of *H. armigera* larvae to both terpenoid and green leaf volatiles enhance larval tolerance to the insecticide methomyl. Exposure of the fifth instars to two terpene volatiles limonene and nerolidol, and two green-leaf volatiles 2-heptanone and *cis*-3-hexenyl acetate all dramatically reduced larval susceptibility to the insecticide methomyl (Figure 1). However, pretreatment with PBO, a well-known P450 inhibitor, compromised the effects of volatile exposure, leading to more susceptibility of *H. armigera* larvae to the insecticide (Figure 1). This finding suggests that insects have the capacity to use plant HIPVs as olfactory cues to upregulate cytochrome P450 monooxygenases (P450s) for detoxification of insecticides.

Plants synthesize thousands of diversified secondary metabolites that act as defensive agents (allelochemicals) against herbivorous insects. Insect pests have frequently been exposed to a wide range of natural and synthetic xenobiotics, including insecticides. However, the insects have evolved capacity to enhance detoxification enzyme systems to neutralize host plant chemical defenses and metabolize insecticides [49,50]. In herbivorous insects various enzymes including cytochrome P450s (P450s), carboxylesterases (CaE), glutathione S-transferases (GST), UDP-glycosyltransferases and digestive proteases are involved in detoxification and metabolism of plant defensive compounds and insecticides [49,50,51,52,53]. Among these, cytochrome P450s that can metabolize a broad range of substances are of particular importance for insect adaptation to various chemicals [54]. P450s are believed to be the primary detoxification enzymes for insect resistance to a wide range of synthetic insecticides [55,56]. Many P450s that are involved in allelochemical detoxification are also responsible for insecticide resistance [57]. Expression of insect P450 genes is constitutively low and phytochemical-inducible [58]. More importantly, many insects can utilize these plant defensive compounds as inducers to enhance detoxification capacity [59,60,61]. For example, several P450 genes from the 6B, 321A and 9A subfamilies are induced by plant allelochemicals (indole, indole-3-carbinol, quercetin, 2-tridecanone and xanthotoxin) in the destructive lepidopteran pest *Spodoptera frugiperda*. The polyphagous caterpillar *Helicoverpa zea* deploys host plant coumarin and xanthotoxin to induce its own detoxifying cytochrome P450s *CYP6B8* and *CYP321A1* [60]. In *H. armigera* several cytochrome P450 genes belonging to CYP6 (including *CYP6AE14*, *CYP6B2*, *CYP6B6* and *CYP6B7*) and CYP9 families can be induced by gossypol and xanthotoxin [62]. The expression of *CYP6B7* has been documented to be induced by three insecticides in *H. armigera:* fenvalerate, phoxim and indoxacarb [63]. The induction levels could reach 127.9- and 316.8-fold by fenvalerate and phoxim, respectively in the insecticide-resistant strain.

Our previous study showed that P450 genes *CYP6B2*, *CYP6B6* and *CYP6B7* were induced by the four allelochemicals flavone, visnagin, coumarin and DIMBOA (2,4-Dihydroxy-7-methoxy-1,4-benzoxazin-3-one) in both midguts and fat bodies in *H. armigera* [23]. The induction of P450s by plant allelochemicals and associated increased resistance has been extensively demonstrated in diet allelochemical-supplemented experiments. However, this study indicates that olfactory exposure to HIPVs could enhance insecticide resistance and induce P450s without consumption of the allelochemicals. Three P450 genes *CYP6B2*, *CYP6B6* and *CYP6B7* were induced by both terpene volatiles and two green-leaf volatiles (Figure 2, Figure 3 and Figure 4). Cytochrome P450s *CYP6B6* and *CYP6B7* have been reported to be involved in pyrethroid insecticide resistance [28,64,65]. Overexpression of *CYP6B7* has been found in pyrethroid-resistant populations of *H. armigera* [66]. Furthermore, P450 enzyme activities were induced by all four selected plant volatiles in both midguts and fatbodies (Table 1). Strong induction of these P450 genes and enzyme activities by the four volatiles may contribute to HIPV-induced insecticide tolerance in *H. armigera.* In addition, 2-heptanone and limonene also induced activities of detoxification enzymes glutathione-s-transferase (GST) and carboxylesterase (CarE) (Table 1), suggesting possible involvement of GST and CarE in HIPVs-induced insecticide tolerance. Based on the results that exposure to piperonyl butoxide nullified HIPV-induced insecticide resistance and all four tested volatiles enhanced activities of P450s but only two volatiles enhanced activities of GST and CarE, we inferred that P450s played a greater role in detoxification of the insecticide.

As olfactory perception of volatile emission is likely more rapid in larvae than dietary consumption, the ability to detect and respond physiologically to HIPVs at a distance prior to actual feeding may confer insect larvae more time to preactivate insect detoxification systems prior to the ingestion or contact of insecticides. Given ubiquity of insect exposure to HIPVs in the interaction with host plants, the mechanism we showed here may play a non-negligible role in insect adaptation to synthetic insecticides. Such effects of plant volatiles should be considered in use of plant-derived volatiles for management of insect pests and in metabolic engineering for improvement of plant defense and pollinator attraction.

## 5. Conclusions

In conclusion, olfactory-mediated exposure of *Helicoverpa armigera* larvae to plant volatiles induces cytochrome P450 monooxygenases and enhances larval tolerance to the insecticide methomyl, revealing a novel mechanism for insect herbivores to gain their detoxification capacity and insecticide resistance.

## Figures and Tables

**Table 1:** Activities of glutathione-s-transferase (GST), carboxylesterase (CarE) and cytochrome P450 enzymes (P450s) in midguts and fatbodies of *Helicoverpa armigera* larvae exposed to volatile 2-heptanone (Hept), *cis*-3-hexenyl acetate (Hylac), limonene (Limo) and nerolidol (Nero) (mmol·min^−1^·mg·prot^−1^).

Volatiles	GST		CarEs		P450s	
	Midgut	Fatbody	Midgut	Fatbody	Midgut	Fatbody
**CK**	3.56 ± 0.15 c	1.78 ± 0.32 b	28.55 ± 0.29 b	6.23 ± 0.78 b	0.26 ± 0.02 c	0.21 ± 0.01 c
**Hept**	5.12 ± 0.17 a	2.75 ± 0.04 a	37.45 ± 1.01 a	9.74 ± 0.17 a	0.52 ± 0.01 a	0.79 ± 0.03 a
**Hylac**	2.11 ± 0.19 d	1.82 ± 0.05 b	26.22 ± 1.29 b	5.67 ± 0.15 b	0.44 ± 0.02 a	0.33 ± 0.01 b
**Limo**	4.34 ± 0.24 b	2.55 ± 0.04 a	28.78 ± 0.59 b	10.97 ± 0.64 a	0.44 ± 0.03 a	0.38 ± 0.01 b
**Nero**	3.68 ± 0.12 bc	1.85 ± 0.25 b	28.00 ± 0.33 b	5.36 ± 0.66 b	0.35 ± 0.02 b	0.32 ± 0.03 b

Newly molted fifth instars were individually exposed to volatile 2-heptanone (Hept), cis-3-hexenyl acetate (Hylac), limonene (Limo) and nerolidol (Nero) at a dose of 1 µL per cup for 48 h in a sealed cup. Then midguts and fat bodies were dissected for enzyme assay. Control larvae (CK) were fed with diet without plant volatile. Data shown are means ± SE derived from three biological repeats (*n* = 3). Different letters above bars indicate significant differences (*p* < 0.05 using ANOVA followed by a Tukey test for post-hoc comparison).

## Supplementary Materials

The following are available online at https://www.mdpi.com/2075-4450/12/3/238/s1. Table S1: The primers forward and reverse sequences used for quantitive RT-PCR.
