# Supplementary material for: Exposure of Helicoverpa armigera Larvae to Plant Volatile Organic Compounds Induces Cytochrome P450 Monooxygenases and Enhances Larval Tolerance to the Insecticide Methomyl
Source: Insects. 2021 Mar 12;12(3):238. doi: 10.3390/insects12030238 (PMC7998352; doi:10.3390/insects12030238)
Supplement: Supplementary file 1 [file insects-12-00238-s001.pdf]

Table S1. The primers forward and reverse sequences used for quantitive RT-PCR

Gene Name (Accession number)		Sequence	Length (mer)	Product Length (bp)	Tm （℃）
CYP6B2 (U18085)	-F	5’-tgatggagacacttggaggactt -3’	23	139	52
	-R	5’-atgcttttcacactctttgctcac -3’	24		
CYP6B6 (AF031468)	-F	5’- tcttgtggacaacattattagc -3’	22	130	59.4
	-R	5’-aagtgatgttactccatcaaga -3’	22		
CYP6B7 (AF031468)	-F	5’-tcttgtggacaacattattagc -3’	22	130	52
	-R	5’-aagtgatgttactccatcaaga-3’	22		
	-R	5’-gtctcggtggaaggcaaaag -3’	20		
EF-1a (U20129)	-F	5’- gacaaacgtaccatcgagaag -3’	21	279	51.1
	-R	5’- gataccagcctcgaactcac -3’	20		
